# The Role of Exogenous Methyl Jasmonate in the Morphophysiology and Postharvest Attributes of Drought-Stressed Radish

**DOI:** 10.3390/plants15030397

**Published:** 2026-01-28

**Authors:** Damiana J. Araujo, Vanessa A. Soares, Estephanni F. O. Dantas, Antônio N. Andrade, Cosma J. Araujo, Daniel S. Gomes, Sabrina K. Santos, Adriano S. Lopes, José E. S. Ribeiro, Valquiria C. S. Ferreira, Juliane M. Henschel, Tancredo Souza, Thiago J. Dias, Diego S. Batista

**Affiliations:** 1 Programa de Pós-Graduação em Ciências Agrárias (Agroecologia), Universidade Federal da Paraíba, Bananeiras 58220-000, PB, Brazil; damianaaraujo18@gmail.com (D.J.A.); tancredo_agro@hotmail.com (T.S.); 2 Programa de Pós-Graduação em Agronomia, Universidade Federal da Paraíba, Areia 58397-000, PB, Brazilthiagojardelinodias@gmail.com (T.J.D.); 3 Departamento de Agricultura, Universidade Federal da Paraíba, Bananeiras 58220-000, PB, Brazil; 4 Departamento de Engenharia de Alimentos, Universidade Federal da Paraíba, João Pessoa 58033-455, PB, Brazil; 5 Departamento de Gestão e Tecnologia Agroindustrial, Universidade Federal da Paraíba, Bananeiras 58220-000, PB, Brazil

**Keywords:** bioregulator, drought tolerance, *Raphanus sativus* L., storage root quality, water stress

## Abstract

Radish is a nutrient- and antioxidant-rich root vegetable whose growth is strongly affected by water availability, highlighting the need for strategies to enhance drought tolerance. Methyl jasmonate (MeJa) is a bioregulator involved in plant stress responses. This study evaluated the role of MeJa in alleviating water deficit effects in radish. Plants were maintained under well-watered conditions (80% water retention capacity) or subjected to total irrigation restriction from 15 to 30 days after sowing (DAS). Foliar applications of 100 µM MeJa or water were performed at 7, 14, and 21 DAS. Growth, gas exchange, chlorophyll fluorescence, photosynthetic pigments, relative water content, electrolyte leakage, and storage root quality were assessed. Water deficit reduced relative water content and increased electrolyte leakage, indicating oxidative damage, which impaired growth and gas exchange. MeJa application reduced electrolyte leakage but did not mitigate drought-induced reductions in growth or gas exchange. Notably, water deficit increased sugar, mineral, and antioxidant contents in roots, regardless of MeJa treatment. Overall, although MeJa modulated some stress-related physiological responses, enhancing antioxidant defenses, it was insufficient alone to improve drought tolerance in radish.

## 1. Introduction

Abiotic stresses are a major threat to agriculture, with water deficit being one of the main environmental factors that reduce plant growth and development [1]. Water deficit occurs when the plant evapotranspiration rate is greater than water uptake, being one of the main factors affecting agricultural production worldwide, as it limits plant growth, development, and composition [2,3]. Water deficit affects plant physiology, morphology, and metabolism, manifesting itself through reduced nutrient uptake, water content, leaf water potential, turgor pressure, stomatal conductivity, and cell expansion and proliferation, also reducing the photosynthetic activity and leaf area [4,5]. In addition, it interferes with the production and allocation of photoassimilates [6], causes changes in chlorophyll content, and induces oxidative stress due to the overproduction of reactive oxygen species (ROS) [3,7].

To deal with these detrimental effects, plants induce defense responses to increase osmoregulation and water uptake from the soil, reduce water loss through stomata, and improve antioxidant defenses [5,8]. Among these responses are the molecular signals that induce adjustments in root morphology following hydrotropism, accumulation of compatible osmolytes, stomatal closure, membrane remodeling, and the synthesis of enzymatic and non-enzymatic antioxidants [9,10,11]. Another aspect related to drought stress is its effect on plant composition. For example, water deficit is known to increase the contents of several bioactive compounds in vegetables, such as phenolics, anthocyanins, betalains, sugars, and proline, increasing their antioxidant capacity and postharvest quality [12,13,14].

In radish (*Raphanus sativus* L.), an important root vegetable, water deficit is known to strongly impair root yield [15]. Radish belongs to the Brassicaceae family and is cultivated and consumed worldwide [16,17]. It is a small root vegetable with a variety of colors, ranging from white to purple and black, with generally white pulp [18]. It has a short cycle and is generally used in salads, as it is rich in nutrients and antioxidants, such as phenolic compounds, anthocyanins, carotenoids, and ascorbic acid [19,20,21]. In addition to its economic importance, the fast growth rate and high plasticity in response to environmental conditions allow radish to be a good model for studying plant growth and production under abiotic stress conditions, such as drought, since under such conditions this species tends to allocate less mass to roots [2,22]. The effects of water deficit in radish include reduced biomass production in leaves and storage roots, increased leaf thickness and greenness and shoot/root ratio, and altered anatomical aspects of leaves [2,23]. Moreover, water deficit induces stomatal closure, limiting CO_2_ uptake and decreasing photosynthesis, also increasing electrolyte leakage and reducing relative water content [15,24,25]. Regarding radish composition, the effects of water deficit remain elusive. While some studies report that water deficit modulates radish composition by increasing the concentration of proline, glycinebetaine, total sugars, sucrose, tocopherol, phenolics, and flavonoids and the activities of antioxidant enzymes [23,25,26,27], other studies report that drought decreases the concentration of phenolics [28] or does not affect radish composition at all [2]. This discrepancy raises the need for further investigating the effects of water deficit in radish composition as well as its interaction with potential stress mitigators.

Considering the strong detrimental effects of water deficit in vegetable production, the search for strategies to improve tolerance is a main objective. These strategies include irrigation, mulching and fertilizing practices, sowing time, selection of tolerant genotypes, the use of soil amendments that increase soil water content, inoculation with growth-promoting microorganisms, and exogenous application of osmoprotectants, plant biostimulants, and phytohormones [3,29]. Methyl jasmonate (MeJa) is a phytohormone belonging to the jasmonic acid group, widely found in plants [30]. Its synthesis occurs through the octadecanoic pathway, which involves a series of metabolic steps after the oxidation of linolenic acid [31]. It is a cellular bioregulator that plays an important role in several plant development processes, from seed germination to senescence [32]. Furthermore, methyl jasmonate has been identified as a regulator that plays a crucial role in mediating several biochemical and physiological processes related to defense and response against biotic and abiotic stresses [33,34], playing a key role in increasing the activity of antioxidant enzymes [35].

Under water deficit, methyl jasmonate is known to play a significant role in mitigating its adverse effects. Although the exact mechanisms by which MeJa mitigates drought stress have yet to be described, it is known that drought increases the endogenous level of JA, which in turn modulates the expression of genes and transcription factors involved in defense responses [36,37]. As a result, MeJa induces antioxidant defenses by promoting the activity of antioxidant enzymes and the production of antioxidant compounds (ascorbate, phenolics, and glutathione), osmoregulation by osmolyte accumulation (proline and glycine betaine), and morphophysiological adjustments on growth and photosynthesis by interacting with other hormones, such as ABA and ethylene [36,38]. Taken together, these MeJa-induced responses improve tolerance against drought by: (1) decreasing oxidative damage to membranes and cell components by detoxifying ROS through antioxidant mechanisms; (2) improving water balance by osmoregulation adjustments and stomatal closure, preventing plant desiccation and improving water use efficiency; (3) photochemical and biochemical adjustments that improve the photosynthetic capacity; and (4) growth modulation to optimize plant survival, such as reduced root growth [36,37,39].

The role of MeJa in inducing water deficit tolerance has been demonstrated by research conducted with cauliflower (*Brassica oleracea* L.), soybean (*Glycine max* L.), and sugar beet (*Beta vulgaris* L.) [40,41,42]. Similarly, in wheat and purple basil plants subjected to drought, exogenous application of MeJa increased antioxidant capacity, improving photosynthesis and maintaining plant growth and yield [43,44,45]. In radish, exogenous MeJa is reported to increase the content of phenolics, anthocyanins, and glucosinolates, resulting in high antioxidant capacity in the absence of drought [46,47]. Despite the evidence suggesting that MeJa can improve antioxidant defenses in radish, no studies have explored the potential of exogenous MeJa in drought stress mitigation in this species so far. Moreover, considering that MeJa is reported to increase the production of antioxidant compounds that confer health-promoting effects to vegetables such as radish, exogenous MeJa may also represent a strategy to improve its postharvest quality, adding value and increasing the commercial appeal of roots. Thus, investigating foliar MeJa treatment in water deficit mitigation of radish will provide important knowledge regarding drought tolerance mechanisms in root crops as well as pave the way for cultivation strategies that can improve food production and quality in a global climate change scenario. In this sense, the objective of this research was to evaluate the effect of exogenous applications of methyl jasmonate on the morphophysiology and postharvest quality of radish plants under water deficit. We hypothesize that MeJa mitigates drought stress in radish by modulating their water relations, photosynthesis, and growth and by improving antioxidant defenses, which in turn improves the postharvest quality of roots.

## 2. Results

**Figure 1:**
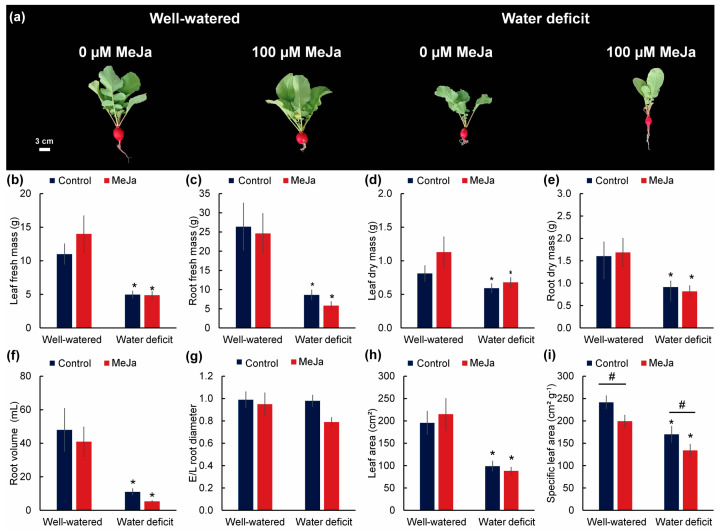
Morphological parameters of 30-day-old radish plants sprayed with water (control) or methyl jasmonate (100 µM MeJa) and grown under irrigation with 80% water holding capacity (well-watered) or water restriction for 15 days (water deficit). (**a**) Plant phenotype; (**b**) leaf fresh mass (g); (**c**) root fresh mass (g); (**d**) leaf dry mass (g); (**e**) root dry mass (g); (**f**) root volume (mL); (**g**) equatorial-to-longitudinal root diameter ratio; (**h**) leaf area (cm^2^); (**i**) specific leaf area (cm^2^ g^−1^). Values represent means ± standard error (*n* = 10). Asterisks (*) indicate differences between irrigation levels, and hashtags (#) indicate differences between methyl jasmonate concentrations by F test (*p* ≤ 0.05).

The growth of radish plants was drastically reduced by water deficit compared with well-watered plants (Figure 1a). For all variables in which a significant effect of at least one of the factors (irrigation regime and methyl jasmonate application) was detected, a significant interaction between these factors was also observed. In plants not treated with MeJa, water deficit reduced the fresh and dry weight of leaves by 54.73% and 27.09%, respectively, and the fresh and dry weight of roots by 67.32% and 42.98%, respectively (Figure 1b–e). Water deficit also reduced root volume by 81.57%; however, the equatorial diameter/longitudinal diameter ratio of roots was not affected by any of the factors (Figure 1f–g). Regarding leaf area, there was a reduction in plants under water deficit and no effect of methyl jasmonate treatment (Figure 1h), whereas specific leaf area was higher in plants under water deficit and lower in plants treated with methyl jasmonate (Figure 1i).

**Figure 2:**
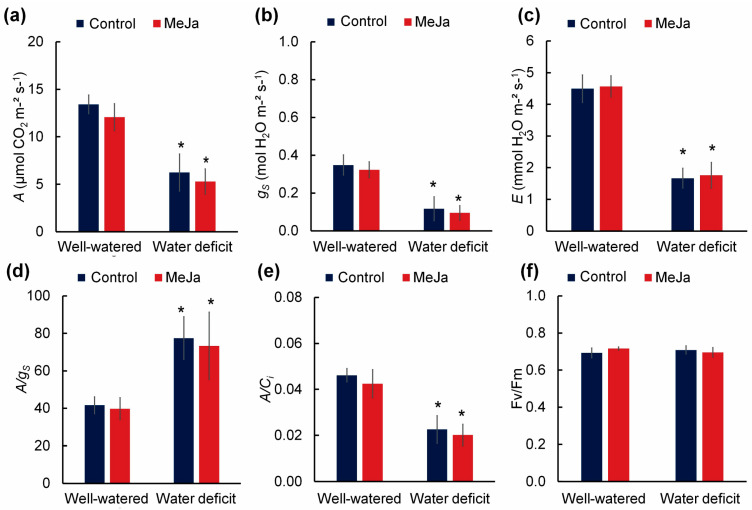
Gas exchanges and chlorophyll fluorescence of 27-day-old radish plants well-watered (80% water holding capacity) or under water restriction for 15 days (water deficit). Plants were sprayed with water (control) or 100 µM methyl jasmonate (MeJa). (**a**) Net carbon assimilation rate (A; µmol CO_2_ m^−2^ s^−1^); (**b**) stomatal conductance (gs; mol H_2_O m^−2^ s^−1^); (**c**) evapotranspiration rate (E; mmol H_2_O m^−2^ s^−1^); (**d**) intrinsic water use efficiency (A/gs); (**e**) carboxylation efficiency (A/Ci); (**f**) maximum quantum yield of photosystem II (Fv/Fm). Values represent means ± standard error (*n* = 10). Asterisks indicate differences between irrigation levels by F test (*p* ≤ 0.05).

Water deficit reduced CO_2_ assimilation (A), stomatal conductance (gs), evapotranspiration (E), and carboxylation efficiency (A/Ci), while increasing the intrinsic water use efficiency (A/gs) by 86.12% (Figure 2a–e). The maximum quantum yield of PSII (Fv/Fm) was not affected by any of the factors (Figure 2f). MeJa treatment, in turn, did not affect any of the gas exchange variables.

**Figure 3:**
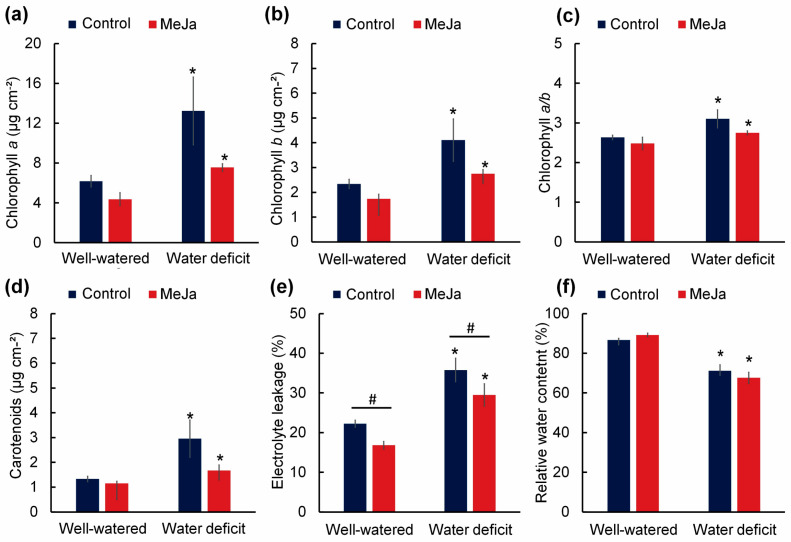
Photosynthetic pigment contents, electrolyte leakage, and relative water content in 27-day-old radish plants well-watered (80% water holding capacity) or under water restriction for 15 days (water deficit). Plants were sprayed with water (control) or 100 µM methyl jasmonate (MeJa). (**a**) Chlorophyll a (µg cm^−2^); (**b**) chlorophyll b (µg cm^−2^); (**c**) chlorophyll a/b ratio; (**d**) carotenoids (µg cm^−2^); (**e**) electrolyte leakage (%); (**f**) relative water content (%). Values represent means ± standard error (*n* = 10). Asterisks (*) indicate differences between irrigation levels, and hashtags (#) indicate differences between methyl jasmonate concentrations by F test (*p* ≤ 0.05).

Pigment concentrations were affected only by irrigation and were not affected by MeJa (Figure 3). The amounts of chlorophyll a, chlorophyll b, and carotenoids, as well as the chlorophyll a/b ratio, were higher in plants under water restriction by 114.36%, 75.64%, 17.73%, and 121.93%, respectively, compared with well-watered plants (Figure 3a–d). Electrolyte leakage was affected by both irrigation and MeJa, while relative water content was affected only by irrigation (Figure 3e,f). There was an increase in electrolyte leakage by 60.84% in radish plants not treated with MeJa and under water deficit conditions compared with the well-watered control; the application of methyl jasmonate was effective in reducing electrolyte leakage (Figure 3e). Furthermore, water deficit reduced the relative water content of radish plants (Figure 3f).

**Figure 4:**
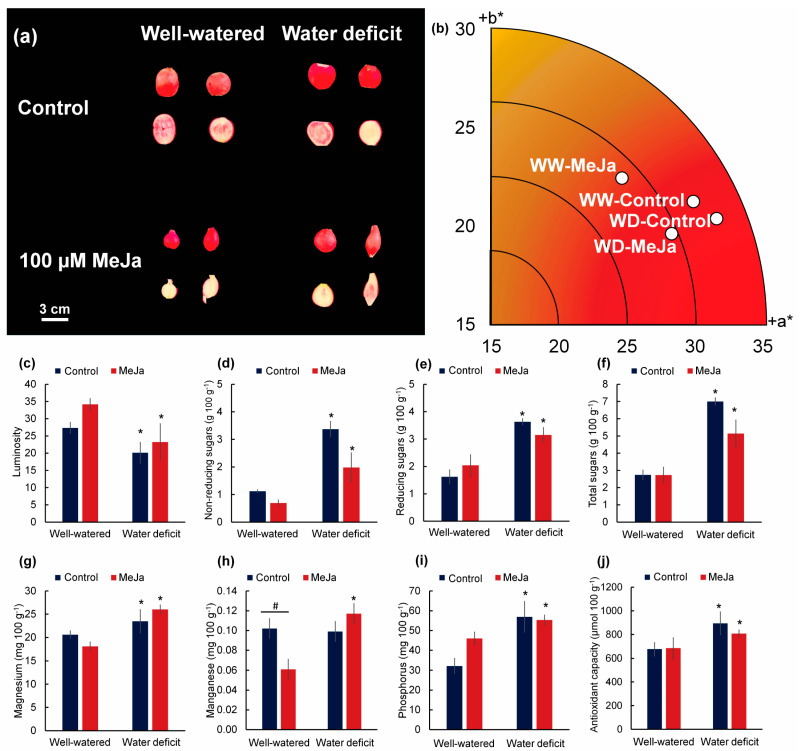
Color parameters, sugar contents, and antioxidant capacity in 30-day-old radish storage roots grown under well-watered conditions (80% water holding capacity) or under water restriction for 15 days (water deficit). Plants were sprayed with water (control) or 100 µM methyl jasmonate (MeJa). (**a**) Representative images of radish roots, showing their skin and pulp; (**b**) distribution of treatments in the CIELAB color space; (**c**) luminosity (L*); (**d**) non-reducing sugars (mg 100 g^−1^); (**e**) reducing sugars (mg 100 g^−1^); (**f**) total sugars (mg 100 g^−1^); (**g**) magnesium (mg 100 g^−1^); (**h**) manganese (mg 100 g^−1^); (**i**) phosphorus (mg 100 g^−1^); (**j**) ABTS antioxidant capacity (µg 100 g^−1^). Values represent means ± standard error (*n* = 10). Asterisks (*) indicate differences between irrigation levels, and hashtags (#) indicate differences between methyl jasmonate concentrations by F test (*p* ≤ 0.05).

Among the color variables evaluated, the parameters a* and b* were not affected by any of the factors, with all treatments being located close to the +a* axis, revealing a tendency toward a reddish color (Figure 4a,b). On the other hand, luminosity (L*) was affected by irrigation, but not by MeJa, and radish roots under water deficit presented lower luminosity (L*) compared with well-irrigated plants (Figure 4c). The irrigation factor also affected the sugar and mineral contents of radish roots. In plants under water deficit, not treated with MeJa, reducing and non-reducing sugars increased by 124.07% and 200.89%, respectively, compared with well-irrigated plants (Figure 4d–f).

Regarding minerals, there was an increase in magnesium and phosphorus concentrations in the roots of plants under water deficit, while manganese concentration was affected by both irrigation and MeJa (Figure 4g–i). In plants under water deficit, magnesium and phosphorus increased by 13.88% and 76.94%, respectively, compared with the well-watered control condition, while well-watered plants treated with MeJa showed a 40.20% reduction in manganese concentration compared with the well-watered control condition. Manganese, zinc, calcium, magnesium, and phosphorus presented values of 0.102, 1.51, 41.69, 20.6, and 32.17 mg 100 g^−1^, respectively, in well-watered and non-MeJa-treated plants, with calcium being the mineral found in a greater quantity under this condition. In this study, the concentration of zinc and calcium in the roots did not show any difference between the treatments and the values of copper and iron in the roots were found at concentrations below the detectable level.

The antioxidant activity, determined by the DPPH and FRAP methods, was not affected by any of the factors; however, in the ABTS method, the untreated radish roots grown under water deficit had an increase of 32.21% in antioxidant capacity compared with the well-watered plants (Figure 4j). The phenolic content did not show differences between treatments.

## 3. Discussion

Plant growth is one of the processes most sensitive to drought [2], and water deficit is one of the factors that contribute most to production losses in vegetable crops [48]. This occurs because cell expansion and division, which fuel plant growth, depend on cell turgor pressure, which in turn requires good water availability [49]. In addition, the stress caused by water deficit reduces stomatal opening, leading to a decrease in CO_2_ uptake and interruption of photosynthesis, a fundamental process for plant development [4]. In this study, radish plants grown under water deficit had lower relative water content and, consequently, lower growth, biomass accumulation, and leaf area. The growth reduction was followed by decreased CO_2_ assimilation rates and stomatal conductance. Root growth is an important plant response to water availability, which can be directed through hydrotropism to explore other soil areas and reach water [50,51]. In species with roots as their storage organs such as radish, water deficit is reported to impact mainly the storage roots [2]. This occurs because the source organs are unable to maintain an adequate supply of assimilates to support the growth of other organs under such conditions [6]. In this sense, the reduction in radish root growth observed here may be related to the reduction in leaf area. Specific leaf area is the surface area of light capture per unit of dry matter [52]. It is a parameter that reflects aspects of leaf morphology, such as leaf thickness, through carbon allocation [52,53]. Here, the increase in specific leaf area under water deficit indicates an increase in leaf thickness as an acclimatization strategy to stress conditions, but, when methyl jasmonate was applied, there was a reduction in specific leaf area.

Chlorophyll a and b are essential pigments that act in the direct absorption of light, while carotenoids act in the photoprotection of the photosynthetic apparatus against excessive light and oxidative damage, often related to the presence of ROS [54]. In radish plants under water deficit, there was an increase in the concentrations of chlorophyll a, chlorophyll b, and carotenoids. This result may be related to the reduction in the leaf area of radish plants. Similar results were also observed in other studies with radish [2], red beet [55], and potato [56]. Despite the reported role of MeJa in stimulating chlorophyll biosynthesis [36], in this study no effects were observed in chlorophyll and carotenoid concentration.

Chlorophyll a and b are the main pigments involved in light absorption, whereas carotenoids play a key role in the photoprotection of the photosynthetic apparatus, particularly under conditions associated with oxidative stress and reactive oxygen species (ROS) accumulation [54]. In radish plants subjected to water deficit, the concentrations of chlorophyll *a*, chlorophyll *b*, and carotenoids increased. This response may, at least in part, be associated with a concentration effect resulting from reduced leaf area under drought conditions. Similar increases in pigment concentrations under water deficit have been reported in radish [2], red beet [55], and potato [56]. Although methyl jasmonate (MeJa) has been reported to stimulate chlorophyll biosynthesis in other species [36], no significant effects of MeJa on chlorophyll or carotenoid concentrations were observed in the present study. However, alternative or complementary explanations, such as drought-induced adjustments in pigment metabolism, altered degradation rates, or changes in chloroplast organization, cannot be excluded.

Stomatal closing is a strategy used to avoid water loss; however, it limits photosynthesis due to the decrease in internal CO_2_ [57]. Here, water deficit drastically reduced gs, resulting in lower A and E. In addition, the instantaneous carboxylation efficiency (A/Ci) of radish plants was affected by water deficit, while Fv/Fm was not, pointing to biochemical damage, while methyl jasmonate was not able to mitigate these effects. Intrinsic water use efficiency, understood as the ratio of net photosynthetic rate to stomatal conductance, plays an important role in quantifying carbon uptake and water loss and provides information on the mechanisms of plant physiological responses to climate change [58,59]. MeJa is reported to decrease water loss in drought-stressed plants by inducing stomatal closure [37,38]. It works by interacting with calcium, H_2_O_2_, and ABA and by modulating the expression of JASMONATE-ZIM-DOMAIN (JAZ), PROTEIN PHOSPHATASE 2C1 (SlPP2C1), and REGULATOR 26, genes related to stomatal movements, resulting in higher water use efficiency [37,39]. However, here, radish plants increased their water use under water deficit independently of MeJa treatment, pointing to the great resilience of this species under this condition and to the lack of an effect of MeJa in the water-saving mechanisms of radish. This is further supported by our relative water content results, showing that MeJa did not affect this characteristic.

Electrolyte leakage is widely used as an indicator of cell membrane integrity, with higher values reflecting increased membrane permeability and ion efflux [60]. Under water deficit conditions, membrane damage is often associated with oxidative stress, which can compromise plasma membrane stability [44]. In the present study, water deficit increased electrolyte leakage, indicating impaired cell integrity, whereas methyl jasmonate (MeJa) application significantly reduced electrolyte leakage, suggesting improved membrane stability as a primary physiological response to MeJa treatment. Similar reductions in electrolyte leakage following MeJa application have been reported in *Triticum aestivum* under stress conditions [44]. Although MeJa has been associated in previous studies with the activation of antioxidant systems and the accumulation of osmoprotective compounds [7,37,42], no direct measurements of antioxidant enzyme activities, reactive oxygen species levels, or lipid peroxidation were performed here. Therefore, the involvement of antioxidant-mediated mechanisms in the MeJa-induced reduction in electrolyte leakage warrants further experimental validation.

In plants under adverse conditions, the accumulation of antioxidants, such as ascorbic acid and phenolic compounds, and osmoprotectants, such as some sugars and amino acids, represents a defense mechanism against the effects of stress [5,61]. The increase in total sugars in the roots of radish plants under water deficit suggests an osmotic adjustment process through the increase in solutes inside the cells. Under drought conditions, these compounds, in addition to helping the osmotic balance of the plant, facilitating the retention and absorption of water in conditions of low availability, also regulate antioxidant activity, contributing to the elimination of ROS [62]. The increase in total sugars may also imply a reduction in the synthesis of structural sugars, thus reducing plant growth. On the other hand, the higher concentration of these compounds increases the quality of the radish, contributing to a better flavor and bringing benefits to the health of the consumer. Moreover, water deficit and methyl jasmonate are known to activate antioxidant defense mechanisms, which include antioxidant enzymes (catalase, peroxidase, and superoxide dismutase) and compounds (ascorbic acid, phenolics, flavonoids, anthocyanins, etc.) [63,64].

Here, water deficit increased the contents of sugars and antioxidant capacity in radish roots, while no effects were found in phenolic compound contents. On the other hand, methyl jasmonate did not affect the contents of sugars and phenolics and the antioxidant capacity in roots. Considering the widely known role of MeJa in promoting antioxidant defense [37], its lack of an effect in inducing the production of antioxidant compounds observed here suggests that MeJa may be increasing membrane stability through other ways than those measured here, such as by increasing the activity of catalase, peroxidase, and superoxide dismutase, and that the antioxidant capacity found in roots may not correlate to that observed in leaves. It is also important to note that the substrate used in this experiment had an acidic pH (5.0). Such acidity can contribute to oxidative stress in plants, in part because it facilitates the uptake of higher concentrations of heavy metals [65], thereby acting as an additional stressor that may confound the effects attributed solely to water deficit. This aspect is particularly relevant considering that soils in Brazil are generally moderately to strongly acidic [66]. Nevertheless, plants growing in acidic soils often exude organic acids from their roots, a strategy that can help mitigate oxidative stress by reducing the generation of reactive oxygen species (ROS) typically induced under abiotic and biotic stresses [67]. Given these interacting factors, future studies should consider controlling or standardizing substrate acidity to better isolate drought effects and strengthen the interpretation of physiological and biochemical responses.

Luminosity (L*) is a color scale that ranges from black (0) to white (100). Low L* values represent darker colors and higher values represent lighter colors [68]. Here, the lower L* values observed in radish roots under water deficit indicate that water restriction affected this parameter, making the color of the roots more opaque/dull, a characteristic that makes the organ less attractive to the consumer. There was no difference in color (ΔE) between treatments and conditions.

In general, radish roots are a good source of minerals such as calcium, magnesium, and manganese [18]. The higher concentration of these minerals under water deficit may be related to the reduction in root size, which may result in a concentration effect. In this study, the manganese and magnesium values under control conditions are close to those found by Goyeneche et al. [19], while the other minerals showed distant values.

As MeJa is reported to function in apoptosis-like cell death [69], the range in its alleviating role and toxic effects may be closely related to the concentration used and may also vary according to species. For instance, MeJa was reported to efficiently induce drought tolerance in cauliflower and beet at 10 µM [40,42], soybean at 20 µM [41], wheat at 0.25, 100, and 500 µM [43,44], and basil at 1 mM [45]. Conversely, 5 to 500 µM had no effect inducing salt stress tolerance in radish, with 5 mM MeJa even inhibiting growth independently of the stress [70]. Moreover, as drought stress itself can induce endogenous MeJa biosynthesis and accumulation, exogenous application may lead to excessive MeJa levels, potentially masking beneficial effects or even triggering hypersensitive responses. Although 100 µM MeJa improved membrane stability under drought, it did not alleviate the pronounced growth inhibition observed. Importantly, the drought treatment applied here (complete irrigation withdrawal for 15 days) resulted in severe stress, as evidenced by more than an 80% reduction in root volume and substantial biomass loss. Under such severe drought conditions, fundamental metabolic and developmental processes are likely to be strongly impaired, which may limit the capacity of regulatory compounds such as MeJa to exert protective or growth-promoting effects. It is therefore plausible that the applied stress intensity exceeded the regulatory threshold at which MeJa can effectively mitigate drought damage. Under more moderate drought conditions, or at lower MeJa concentrations, different or more pronounced responses might be observed. This distinction between moderate and severe drought stress should be considered when interpreting the lack of growth-related mitigation in the current study and highlights the need for future work addressing both stress intensity and dose–response relationships. Interestingly, MeJa was also reported to be ineffective in inducing tolerance against salt stress in radish, with high concentrations (5 mM) strongly inhibiting radish growth independently of the stress [70]. Thus, future studies might focus on testing the effectiveness of lower MeJa concentrations in drought stress alleviation in radish and the evaluation of endogenous MeJa concentrations and other antioxidative mechanisms. Also, considering the high variability among genotypes of this species, studies using different radish varieties would be valuable to enhance the applicability and generalization of the results.

## 4. Materials and Methods

### 4.1. Experimental Site and Growth Conditions

The experiment was conducted from March to April 2023 in the experimental area in a greenhouse at the Seedling Production Laboratory of the Center for Human, Social, and Agrarian Sciences of the Federal University of Paraíba (CCHSA/UFPB) in Bananeiras, Paraíba, Brazil (6°45 S, 35°38 W, altitude of 526 m). Radish seeds (*Raphanus sativus* L.) of the Crimson Gigante variety (TopSeed^®^, Sarno, Italy) were sown in 5 L polyethylene pots containing a commercial substrate composed of 60% bioactivated pine bark, 15% fine vermiculite, 15% superfine vermiculite, and 10% humus (Mecplant^®^-HORTA 1, Telêmaco Borba, Brazil), with the addition of 5 g of a granulated mixture of nitrogen, phosphorus, and potassium (NPK, 4-14-8). A chemical characterization of the substrate is presented in Table 1.

The pots used were 19 cm in height and 23 cm in width, with 12 holes (≈1 cm diameter) at their bottom to allow for water drainage. Five days after sowing, the plants were thinned, homogeneous plants were selected, and two plants per pot were maintained. All plants were irrigated with 100% of the water retention capacity (WRC), once a day, until the 7th day after sowing (DAS) and then irrigated with 80% of the WRC until the 14th DAS every two days. After that, plants were submitted to two irrigation levels (well-watered or water deficit) as described below. WRC was determined following the lysimetric weighing method [71]. This method consists of weighing the pots containing the dried substrate and then weighing the pots with the substrate saturated with water. The difference between both weights corresponded to the amount of water required to reach 100% of the WRC, of which 80% WRC was estimated. Then, pots were weighed prior to each irrigation, and water was added until the weight corresponding to 80% WRC was reached.

### 4.2. Experimental Design and Treatment Application

The experimental design used was completely randomized in a 2 × 2 factorial scheme (soil moisture levels × bioregulator treatment) with 10 replications. Soil moisture levels corresponded to well-watered or water deficit conditions, while bioregulator levels corresponded to the control (water) or 100 µM methyl jasmonate [70]. As mentioned above, water deficit was imposed from 15 to 30 DAS by total irrigation restriction, while well-watered plants received irrigation with 80% WRC during the entire experimental period. At 7, 14, and 21 DAS, 10 mL of water (control) or 100 µM methyl jasmonate was applied per plant as a foliar spray [70]. The control and methyl jasmonate solutions were prepared using the surfactant polysorbate 80 (Tween-80^®^, 0.03%) (*v*/*v*). Physiological analyses were performed at 27 DAS, while morphological analyses were performed after plant harvesting at 30 DAS. Moreover, after harvesting, radish storage roots were cleaned, ground, and stored at −80 °C until biochemical analyses.

### 4.3. Gas Exchange, Photosynthetic Pigment, and Chlorophyll Fluorescence Measurements

All the following analyses were performed in fully expanded leaves. Gas exchange measurements were performed at 27 DAS, at 8:00 a.m., with an infrared gas analyzer (IRGA—LCpro-SD Portable Photosynthesis Measurement System, ADC BioScientific, Hoddesdon, UK). The reference CO_2_ concentration and temperature were maintained as the ambient, and photosynthetically active radiation of 1000 µmol photons m^−2^ s^−1^ with 10% blue light was used. The net CO_2_ assimilation rate (A), stomatal conductance (gs), transpiration rate (E), water use efficiency (WUE, A/E), internal carbon concentration (Ci), intrinsic water use efficiency (WUEi, A/gS), and instantaneous carboxylation efficiency (A/Ci) were determined [72].

Chlorophyll a fluorescence was determined in leaves pre-adapted to the dark for 20 min using a portable fluorometer (model OS-30p+, Opti-Sciences, Hudson, NH, USA). Minimum fluorescence (Fo), maximum fluorescence (Fm), and maximum photochemical efficiency of photosystem II (PSII) (Fv/Fm) were determined.

Photosynthetic pigment levels were determined according to Wellburn [73], with modifications. For this, four discs (1 cm^2^) of fully expanded leaves from four plants per treatment were incubated in 7 mL of dimethyl sulfoxide for 48 h in the dark [74]. After this period, the extract was read at 480, 649, and 665 nm using a spectrophotometer (GTA-96 UV-VIS, Global Trade Technology, São Paulo, Brazil). The levels of chlorophyll a and chlorophyll b, chlorophyll a/b ratio, and total carotenoids were determined.

### 4.4. Assessment of Relative Water Content and Electrolyte Leakage

To determine the relative water content, 10 leaf discs (1.0 cm in diameter) were collected from fully expanded leaves of 5 plants per treatment. To obtain fresh mass (FM), the discs were weighed on a precision scale immediately after collection. After weighing, the discs were submerged in distilled water for six hours and after this period they were weighed to obtain turgid mass (TM). They were then dried at 65 °C for 24 h to obtain dry mass (DM) [75]. Water content is expressed as a percentage (%) and was calculated as follows: [(FM-DM)/(TM-DM)] × 100.

Electrolyte leakage was quantified according to Bajji et al. [76]. Ten leaf discs (1 cm^2^) were collected from fully expanded leaves and placed in tubes containing 40 mL of distilled water, where they were kept for 4 h at 25 °C with occasional agitation. After this period, the initial electrical conductivity (EC1) of the extract was determined with a portable conductivity meter (CD-880, Instrutherm, São Paulo, Brazil). Then, the tubes were kept at 90 °C for 2 h and, after this period, the final electrical conductivity (EC2) was determined. Electrolyte extravasation is expressed as a percentage and was calculated according to the following equation: EL (%) = (EC1/EC2) × 100.

### 4.5. Growth Parameters, Root Quality, and Postharvest Analyses

At the end of the experiment (30 DAS), the following growth parameters were determined: root diameter, length, and volume, leaf area, specific leaf area, and fresh and dry mass of leaves and roots. Root diameter, root length, and leaf area were determined using ImageJVersion 1.54i software, and root volume was obtained after immersion in a graduated cylinder, obtaining the difference between the final and initial volumes. To determine fresh mass, leaves and roots were weighed separately on a semi-analytical balance immediately after harvest. To determine dry mass, they were then placed in a forced air circulation oven at 65 °C until they reached constant weight. Specific leaf area was determined by the ratio between leaf area and leaf dry mass. In addition, the ratio between shoot and root was calculated using dry mass data.

After physiological and growth analyses, the firmness and colorimetry of the radish storage roots were analyzed, which were then crushed and stored in a freezer at −80 °C until postharvest quality analyses were performed. Total, reducing, and non-reducing sugars, total phenolic compounds, antioxidant capacity (DPPH, ABTS, and FRAP methods), and minerals were analyzed. Approximately 1 g of fresh material was used for the phenolic, antioxidant capacity, and mineral analyses, while 0.25 g was weighed for sugars.

For colorimetric analysis of the peel of roots, the Colorimeter application (Research Lab Tools) developed for devices with the Android^®^ system [77] was used. Measurements were performed at three points of the equatorial portion of roots, from 5 plants per treatment, to determine the values of luminosity (L), red/green intensity (+/−) (a), and yellow/blue intensity (+/−) (b). The total color differences (delta E (ΔE)) between the L*, a*, and b* of the roots were calculated as follows: ΔE = ((ΔL*)^2^ + (Δa*)^2^ + (Δb*)^2^)1/2 [78].

Root firmness was determined in newtons (N) using a digital penetrometer (PTR-300, Instrutherm^®^, São Paulo, Brazil) with a 3 mm tip. Three measurements were taken per root, in the equatorial portion.

### 4.6. Quantification of Sugars, Antioxidant Activity, and Phenolic Compounds

Sugars, antioxidant activity, and phenolic compounds were determined in root samples obtained after crushing. For this, the entire storage root was crushed, including its peel. Total sugars were quantified using the anthrone method described by Yemm and Willis [79]. Reducing sugars were quantified according to the dinitrosalicylic acid (DNS) method proposed by Miller [80]. Non-reducing sugars were determined by the difference between total sugars and reducing sugars. Glucose was used as a reference to obtain the standard curve.

The determination of antioxidant activity was performed using the DPPH (2,2-diphenyl-1-picrylhydrazyl) and ABTS (2.2 ′azinobis (3-ethylbenzthiazoline sulfonic acid-6)) colorimetric methods and the FRAP (Ferric Reducing Ability Power) assay, and the results are expressed as Trolox equivalent antioxidant capacity (TEAC) in µmol TE for each sample. DPPH and ABTS were determined according to Rufino et al. [81], and FRAP was determined according to Boroski et al. [82]. The content of phenolic compounds was determined according to the spectrophotometric method of Folin–Ciocalteu [83] and is expressed in gallic acid equivalents.

### 4.7. Mineral Nutrient Analysis

For the determination of Zn, Mg, Ca, and Mn, the crushed root samples were digested with a nitro-perchloric mixture (HNO_3_:HClO_4_; 5:2) and then analyzed in an atomic absorption spectrometer with a flame atomizer (model iCE 3500, Thermo Scientific, Cambridge, UK). The minerals were analyzed in atomic absorption mode, using hollow cathode lamps containing these elements as the primary radiation source (Photron, Victoria, Australia), and background correction was performed with a deuterium lamp coupled to the equipment. Standard curves were prepared with standard solutions for each element (Specsol, São Paulo, Brazil). To mitigate chemical interference, lanthanum was added to the mineral solutions until a concentration of 0.1% La was reached in the analysis of Mg and Ca. The instrumental parameters were used according to the manufacturer’s recommendations, and the data were processed using the SOLAAR software version 1.5 (Thermo Scientific, Cambridge, UK). The phosphorus content was analyzed by spectrophotometry in a UV-VIS spectrophotometer (model UV-5100, Metash Instruments, Shanghai, China) according to the methodology proposed by Rangana [84]. Iron and copper were also analyzed in atomic absorption mode, but the levels were below the detection limit.

### 4.8. Data Analysis

The data were tested for homogeneity using Bartlett’s test and for normality with the Shapiro–Wilk test. Subsequently, they were subjected to analysis of variance (ANOVA) using the F test to assess the significance of the individual factors and their interactions (*p* ≤ 0.05). Statistical analyses were performed using the Genes software version 2015.5.0 [85].

## 5. Conclusions

Water deficit markedly reduced gas exchange and growth in radish plants, and, under the conditions evaluated here, the application of 100 µM methyl jasmonate (MeJa) did not mitigate these effects. In addition, MeJa did not influence the postharvest quality of radish roots. Thus, under the specific conditions of this study (Crimson Gigante cultivar, 100 µM MeJa, and severe drought stress), MeJa did not significantly improve drought tolerance in radish plants. Although MeJa-treated plants exhibited reduced electrolyte leakage, this response was not accompanied by improvements in water use efficiency, stomatal regulation, osmoregulation, or photosynthetic performance. Together, these results indicate that, within the experimental framework adopted here, MeJa was ineffective in alleviating the negative impacts of water deficit on radish plants. Considering that the exact mechanisms involved in MeJa-induced defense responses are still unclear, further studies are required to assess the potential of lower MeJa concentrations in the mitigation of water deficit effects in radish, including less severe stress conditions, and assess the expression of genes and activity of antioxidant enzymes.

## Figures and Tables

**Table 1:** Chemical characterization of the substrate used in the experiment.

pH _(H2O, 1:2.5)_	P	Na	H + Al	Al	Ca	Mg	K	C
	mg kg^−1^	cmol_c_ kg^−1^						g kg^−1^
5.00	233.27	14.69	20.62	0	8.9	8.9	97.59	35.25

## Data Availability

The original contributions presented in the study are included in the article. Further inquiries can be directed to the corresponding authors.
